# Association between Sedentary Behavior during Leisure Time and Excessive Weight in Chinese Children, Adolescents, and Adults

**DOI:** 10.3390/nu15020424

**Published:** 2023-01-13

**Authors:** Ya Su, Xueyuan Li, Huilun Li, Jiawei Xu, Mi Xiang

**Affiliations:** 1 School of Nursing, Shanghai Jiao Tong University, Shanghai 200025, China; 2 Health Commission of Shanghai Huangpu, Shanghai 200025, China; 3 School of Public Health, Shanghai Jiao Tong University School of Medicine, Shanghai 200025, China; 4 Eye and Dental Diseases Prevention and Treatment Center of Pudong New Area, Shanghai 200025, China; 5 Minister of Education-Shanghai Key Laboratory of Children’s Environmental Health, School of Public Health, Shanghai Jiao Tong University, Shanghai 200025, China

**Keywords:** sedentary behavior, leisure time, excessive weight, Chinese population

## Abstract

(1) Background: Sedentary behavior is increasing in all age groups and is one of the most common lifestyles that is a risk factor for overweight and obesity; however, few studies have evaluated the impact of leisure-time sedentary behavior on overweight and obesity. This study aims to examine the distribution characteristics of different leisure-time sedentary behaviors and their effects on excessive weight in different age groups in the Chinese population to provide evidence for the development of behavioral interventions. (2) Methods: This study utilizes data from the 2004 to 2011 China Health and Nutrition Survey (CHNS). Participants ranged in age from 6 to 18 years or older and were from China. Weight and height were measured in the physical examination, and the sedentary behavior times during leisure periods were collected by using a questionnaire. Logistic regression models were performed for association analyses. (3) Results: A total of 36,169 participants were included in this study. The impact of screen-based sedentary leisure time on excessive weight is greater in middle-aged and older adults. For middle-aged adults, sedentary time periods spent on the Internet or video games were found to be significantly and positively associated with excessive weight (OR, 1.122, 95% CI, 1.005–1.253). In addition, for older adults, sedentary time periods spent watching television or videos were found to be significantly and positively associated with excessive weight (OR, 1.065, 95% CI, 1.035–1.095). (4) Conclusions: This study helps grasp the target population and provide evidence for the development of behavioral intervention guidelines.

## 1. Introduction

China’s economic and technological boom introduced convenience and lifestyle changes for its citizens, but it has also increased the prevalence of overweight individuals [1]. Among Chinese adults, the overweight rate increased from 37.4% in 2000 to 41.2% in 2014, and obesity prevalence increased from 8.6% in 2000 to 12.9% in 2014 [2]. Numerous studies have shown that overweight and obesity are associated with an increased risk of cardiovascular disease [3], cancer [4], and type 2 diabetes [5]. Besides physical conditions, excessive weight is linked to an increased risk of psychological disorders [6], such as anxiety and depression [7]. Although the risks of overweight and obesity are widely understood, current interventions target obesity exclusively. Nevertheless, the overweight prevalence is three times higher than obesity, underlining the urgent need for research on excessive weight which can provide relevant evidence for developing interventions.

Lifestyles, such as physical activity (PA), sedentary behavior (SB), and energy intake, are vital determinants of the body’s weight [8]. As one of the most common lifestyles today and considering its higher health costs, sedentary behavior has become a significant public health problem worldwide. In China, sedentary times are increasing in all age groups [9], with occupational sedentary, commuting sedentary, and leisure sedentary accounting for a large proportion of all sedentary time periods. Therefore, WHO has recommended reducing SB time as part of its physical activity (PA) guidelines [10]. However, leisure-time SB (LT-SB) is the most important compared to occupational and commuting SBs because of the robust connection to unfavorable health outcomes [11]. In addition, LT-SB is modifiable and avoidable since it is lifestyle-dependent.

In a U.S. survey, the total sitting time significantly increased from 2001 to 2016 among adolescents and adults. The prevalence of leisure-time computer use also increased in all age groups [12]. Studies on sedentary leisure behavior and its impact on different age groups have been conducted in the U.S. [12], Australia [13], and some European countries [14], strongly supporting the establishment of public health strategies and recommendations in these countries. China released the Healthy China 2030 blueprint, aiming to reduce overweight changes by altering lifestyles [15]. However, no studies have examined leisure-time sedentary activities within a large sample to date. Furthermore, there is a paucity of empirical studies on the effects of sedentary leisure time on excessive weight in different age groups. Therefore, analyses of the characteristics of LT-SB and the impact on excessive weight in different age groups using nationally representative data are essential for developing future age-specific public health policies and potential programs.

To address the current gaps, we studied the distribution of leisure-time sedentary behavior and the impact of sedentary behavior on excessive weight in the Chinese population using data from the China Health and Nutrition Survey (CHNS).

## 2. Materials and Methods

### 2.1. Study Population

This study is a secondary analysis of Chinese individuals participating in the China Health and Nutrition Survey (CHNS). The population-based China Health and Nutrition Survey (CHNS) has been investigating the health and nutritional status of individuals in China since 1989. The survey’s population was sampled by utilizing a multistage, random cluster process in nine major provinces. Counties in the nine provinces were stratified by income (low, middle, and high), and a weighted sampling scheme was applied to select four counties in each province randomly. [16,17]. Further information on the survey’s procedures and the rationale of the CHNS is available at http://www.cpc.unc.edu/projects/china (accessed on 30 March 2022). The study’s procedures were approved by the Institutional Review Boards of the University of North Carolina at Chapel Hill and the National Institute of Nutrition and Food Safety, China Center for Disease Control and Prevention, and each participant provided written informed consent [16,17]. After removing all identifiable information of the study’s participants, data were released by the CHNS for public use. Our study’s population included all participants over 5 years of age in the CHNS. The analysis used secondary data from four CHNS waves (2004–2005, 2006–2007, 2008–2009, and 2010–2011) since the measurements of sedentary behavior were consistent in these waves.

### 2.2. Anthropometrics and Weight Status

Weight and height were measured by physical examination at the center. The body mass index (BMI) was calculated by the dividing weight (kilogram) by height (meters) squared. In accordance with the National Health and Family Planning Commission of the People’s Republic of China, BMI-to-age percentile cutoffs were used to categorize children and adolescents aged 6–17 years into three groups: normal (5th–84th percentile), overweight (85th–94th percentile), and obese (≥95th percentile) [18]. For adults (≥18 years), the BMI was classified into normal (18.5 kg/m^2^–23.9 kg/m^2^), overweight (24.0 kg/m^2^–27.9 kg/m^2^), and obese (≥28 kg/m^2^). For the analysis in our study, we combined the overweight and obesity groups into a single excessive weight group. Participants with extreme BMIs—including children and adolescents with a BMI that was less than the 5th percentile and adults with a BMI that was lower than 18.5 kg/m^2^ or greater than 60 kg/m^2^—were excluded due to potential underlying health conditions [12].

### 2.3. Assessment of Leisure-Time Sedentary Behavior

The presence of leisure-time sedentary behavior (LT-SB) was assessed by a single question. Adult participants were asked, “Do you participate in this sedentary activity?” Children aged 6 and older were asked, “Do you participate in this sedentary activity when you are not in school?” Participants who answered “yes” were considered to have LT-SB. The following questions concerning the time of LT-SB were then asked: “How much time do you spend during Monday to Friday?” and “How much time do you spend during Saturday to Sunday?” LT-SB was categorized into 1. watch TV or video (watching TV, videotape, VCDs, DVD, movies, and videos); 2. computer use; 3. gaming (video games and online games); 4. homework (6–17 years); and 5. reading (books, newspapers, and magazines). The workday LT-SB was calculated as 5* the daily average time spent from Monday to Friday. The weekend LT-SB was calculated as 2* the daily average time spent on LT-SB from Saturday to Sunday. The total LT-SB was calculated by summing the workday and weekend LT-SB. Participants who reported a total daily sitting time of >16 h were considered outliers and were therefore excluded [12].

### 2.4. Assessment of Leisure-Time Physical Activities

The presence of leisure-time physical activities (LT-PA) was assessed by a single question. Adult participants were asked, “Do you participate in this physical activity?” Children aged 6 and older were asked, “Do you participate in this physical activity when you are not in school?” Participants who answered “yes” were then asked, “How much time do you spend during Monday to Friday” and “How much time do you spend during Saturday to Sunday”. LT-PA included the following: 1. martial arts (kung fu, etc.); 2. gymnastics, dancing, and acrobatics; 3. track and field (running, etc.); 4. swimming; 5. walking; 6. soccer, basketball, and tennis; 7. badminton and volleyball; and 8. others (ping pong, tai chi, etc.). Workday LT-PA was calculated as 5* the daily average time spent on all eight LT-PA categories from Monday to Friday. The weekend LT-PA was calculated as 2* the daily average time spent on all eight LT-PA categories from Saturday to Sunday. The total LT-PA was calculated by summing workday and weekend LT-PA.

### 2.5. Assessment of Energy Intakes

CHNS used three consecutive 24 h dietary recalls to measure energy intake. Participants recorded all types and amounts of food and beverage items consumed at home and away from home during a 24 h period for three consecutive days. Parents of children younger than 12 years old were asked to recall their children’s food consumption. The energy of each food item is based on the China Food Composition. The daily energy intake for each participant was calculated as the energy intake for the three days divided by 3. Participants who reported more than 6210.5 kcal (>99th percentile) of daily total energy intake were considered outliers and were excluded.

### 2.6. Covariates

Self-reported socio-demographic characteristics included sex, age, and educational level. Participants were divided into age groups, children at 6–11 years old, adolescents at 12–17 years old, young adults at 18–44 years old, middle-aged adults at 45–59 years old, and older adults at 60 years old or older. The covariates that were considered as potential confounders in the models included age (in years), sex, educational level, smoking, energy intake, physical activity, region (urban or rural areas), and survey year. Further details for each variable are available on the CHNS Website (http://www.cpc.unc.edu/projects/china (accessed on 30 March 2022).

### 2.7. Statistical Analyses

Statistical analyses included univariate and multivariate analyses. All data analyses were conducted using IBM SPSS Statistics Version 22.0 (IBM, Armonk, New York, NY, USA). Regarding descriptive statistics, continuous variables were presented using mean and standard deviations, while categorical variables were displayed by frequencies and percentages. In univariate analyses, the BMI trend of different age groups in each survey year, the daily LT-SB time trend of different age groups, and the trends in LT-SB of different age groups in each survey year were analyzed by using a one-way analysis of variance. In multivariable analyses, a sex-stratified multivariable logistic regression analysis adjusting for age, smoking status, educational level, energy intakes, physical activity, region (in urban and rural areas), and survey year was performed to determine the relationship between total LT-SB times and excessive weight. Age-stratified multivariable logistic regression analyses were then performed to determine the associations between LT-SB times and excessive weight. Sex, energy intakes, physical activity, region (in urban and rural areas), and survey year were adjusted for all participants, and educational level and smoking status were further controlled in adults. K-means clustering was used to define LT-SB and lifestyle patterns. In addition, an age-group-stratified multivariate logistic regression analysis was performed. The relationship between total LT-SB times and excessive weight was estimated by deriving odds ratios (ORs) and 95% confidence intervals (CIs) from logistic regression models. *p*-values of <0.05 were considered statistically significant.

## 3. Results

### 3.1. Socio-Demographic Characteristics

Of all the participants aged above 5 years, we excluded individuals with missing or extreme data on LT-SB time, body weight, height, physical activities, energy intakes, and other covariates. A total of 36,169 individuals (mean age 47.29 ± 16.32 years as shown in Table S1) were analyzed, including 513 (1.4%) children, 1286 (3.6%) adolescents, 13,494 (37.3%) young adults, 12,492 (34.5%) middle-aged adults, and 8384 (23.2%) older adults. A total of 36% of individuals were from the urban area, 18,841 (52.1%) individuals were female, 27.6% of adults were current smokers, and 4% were former smokers. The education level of most adults was below high school (68.4%), followed by high school (24.5%) and above high school (11.7%).

### 3.2. Overweight and Obesity Status

**Figure 1:**
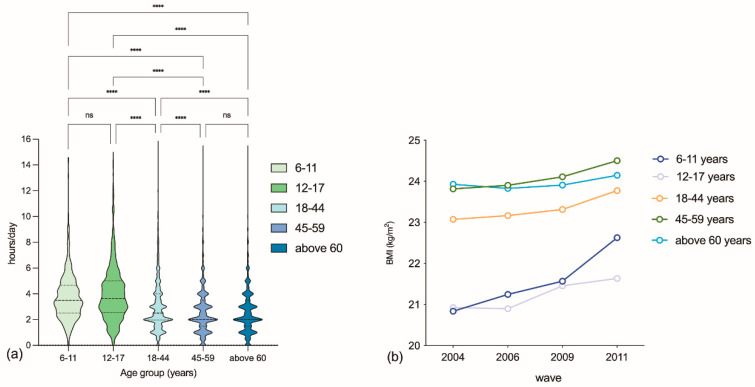
(**a**) Total daily leisure-time sedentary behaviors by age group among the Chinese population, CHNS 2004-2011; (**b**) BMI by age groups and survey years among the Chinese population; ^****^: *p* < 0.0001; ns: not significant.

**Figure 2:**
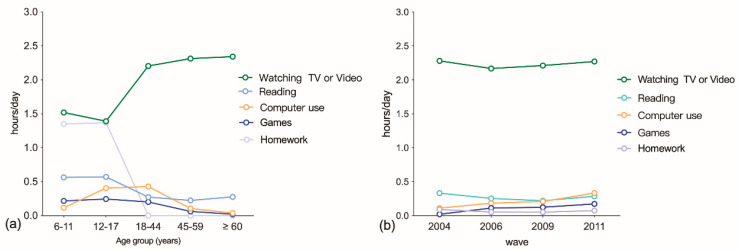
(**a**) Trends in leisure-time sedentary behaviors by age group among the Chinese population, CHNS 2004-2011; (**b**) trends in leisure-time sedentary behaviors by survey years among the Chinese population.

In children and adolescents, the mean BMI was 21.38 ± 2.77 kg/m^2^. In young and middle-aged adults, the mean BMI was 23.79 ± 3.28 kg/m^2^. As shown in Figure 1, there were significant differences in BMI among all age groups, except for the group between 2004 and 2006. The prevalence of obesity and overweight increased in children and adolescents, from 9.9% in 2004 to 23.0% in 2011 (*p* < 0.001), and the prevalence of obesity and overweight increased in adults from 39.1% in 2004 to 47.0% in 2011 (*p* < 0.001). Moreover, the BMI showed an increasing trend in all age groups, as shown in Figure 2. The prevalence of overweight for each age group is presented in Table S2.

### 3.3. Energy Intake and Physical Activity Status

The mean daily energy intake was 2082.11 ± 762.09 kcal in children and adolescents, and 2267.56 ±778.92 kcal in adults. In terms of physical activity, 63.4% of children and adolescents reported less than 60 min per week, followed by 20.8% of the population who reported more than or equal to 300 min per week in leisure time. In adults, 87.8% of the population had less than 60 min of physical activity per week, followed by 8.2% of the population who reported more than or equal to 300 min in leisure time.

### 3.4. Leisure-Time Sedentary Behavior Status

An elevated mean total daily LT-SB time was observed in children and adolescents, from 3.75 ± 1.97 h in 2004 to 4.19 ± 2.05 h in 2011 (*p* = 0.02). Similarly, the mean total daily LT-SB time increased in adults from 2.77 ± 1.98 h in 2004 to 3.08 ± 2.01 h in 2011 (*p* < 0.000). As shown in Figure 1, the total daily LT-SB time in children and adolescents was significantly higher than that in adults. In the overall population, 5923 (16.4%) of the study participants reported a total daily LT-SB time of less than or equal to 1.0 h per day, 10,261 (28.4%) of individuals reported 1.1–2.0 h per day, and 19,985 (55.3%) of individuals reported more than 2.0 h a day. The trends of LT-SB time were different across age groups, with older adults (40.8%) indicating the greatest proportion of individuals who watched TV or video for 2 h or more in their leisure time, followed by middle-aged adults (40.7%) and young adults (38.65%). In contrast, participants who used computers for 1 h or more per day in their leisure time mainly consisted of young adults (19.1%), followed by adolescents (16.9%). Young adults (10.9%) contributed the largest proportion of participants who stayed online or played video games for 1 h or more per day, followed by adolescents (9.0%) and children (7.2%). Only children (67.4%) and adolescents (63.0%) completed homework for 1 h or more per day. However, the population who read for 1 h or more a day mainly comprised children (24.2%), followed by adolescents (21.9%) and older adults (14.8%). From 2004 to 2011, leisure time spent on watching TV or video was the longest in all LT-SB categories, but it tended to stabilize with time. A gradual increase in computer use time was found, as presented in Figure 2. LT-SB, socio-demographic, and lifestyle characteristics by age group are demonstrated in Table S2.

### 3.5. Leisure-Time Sedentary Behaviors Associated with Excessive Weight

**Figure 3:**
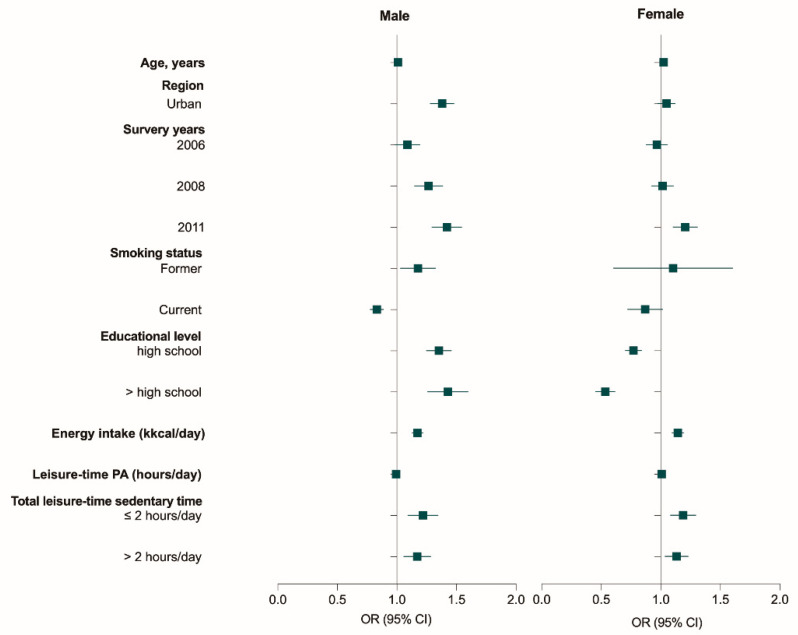
Association between total daily sedentary time and excessive weight among the Chinese population. Note: PA: physical activity; OR: odds ratio; CI: confidence interval; sex, energy intakes, physical activity, region (in urban and rural areas), survey year, educational level, and smoking status were adjusted. Reference for each categorical variable: region: rural; survey year: 2004; smoking status: current; education level: less than high school; total LT-SB time: less than or equal one hour per day.

The total LT-SB time was associated with excessive weight in a sex-stratified multivariate logistic analysis (Figure 3 and Table S3). In both genders, the total LT-SB time above 1 h per day was associated with increased risks of excessive weight after adjusting for age, smoking status, educational level, energy intake, physical activity, region (in urban and rural areas), and survey year. For males who were sedentary for less than or equal to two hours and more than two hours a day, the risk of having excessive weight increased approximately 1.2 times (OR, 1.214, 95% CI, 1.095–1.346; OR, 1.166, 95% CI, 1.059–1.284) than LT-SB time equaled one hour or less a day. Females who were sedentary for less than or equal to two hours and more than two hours a day were 1.2 and 1.1 times (OR, 1.185, 95% CI, 1.084–1.295; OR, 1.130, 95% CI, 1.038–1.230) more likely to have excessive weight than those who were sedentary for one hour or less a day, respectively.

**Figure 4:**
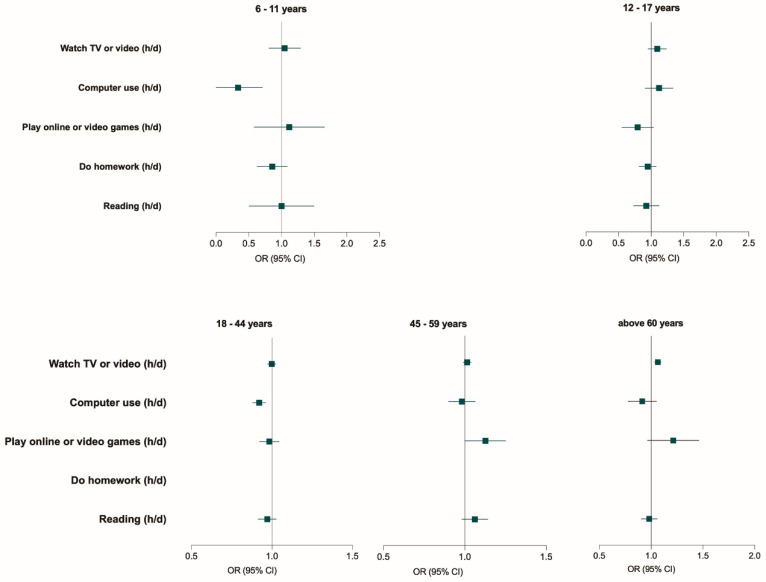
Association between leisure-time sedentary behaviors with excessive weight among the Chinese population, CHNS 2004-2011. Note: OR: odds ratio; CI: confidence interval; h/d: hours per day. For the 6–11- and 12–17-year-old groups, sex, energy intakes, physical activity, region (in urban and rural areas), and survey year were adjusted. For the above 18-year-old group, sex, energy intakes, physical activity, region (in urban and rural areas), survey year, educational level, and smoking status were adjusted.

LT-SB time was associated with excessive weight in age-stratified multivariate analysis (Table 1 and Figure 4). In the 12–17-year group, LT-SBs were not associated with excessive weight in the adjusted model. For children aged 6–11 years and young adults, the LT-SB time of computer usage was negatively associated with excessive weight (OR, 0.200, 95% CI, 0.053–0.755; OR, 0.921, 95% CI, 0.881–0.962). In middle-aged adults, online or video game times significantly increased the risk of excessive weight (OR, 1.122, 95% CI,1.005–1.253). Furthermore, watching TV or video LT-SB times were positively associated with excessive weight (OR, 1.065, 95% CI, 1.035–1.095) in older adults.

## 4. Discussion

The distribution and trends of LT-SB were clarified using a nationally representative sample of the Chinese population in this study. Moreover, the effects of LT-SB on excess weight were examined in all age groups and genders. The prevalence of overweight in the Chinese population remains high, demonstrating an increasing trend of BMI in all age groups from 2004 to 2011. The total daily LT-SB time in children and adolescents was significantly higher than in middle-aged and older adults. Our findings identified the distinct effect of LT-SB on excess weight in different age groups. Thus, this study provides guidance for potential lifestyle and behavioral interventions to improve sedentary behavior and excess weight.

Sedentary time, which is closely related to body weight, increased in Chinese people of all ages [9]. Sedentary behavior leads to a low basal metabolic rate, disrupting the energy expenditure balance and resulting in weight gain [19]. Sedentary times and elevated BMI are major risk factors of noncommunicable diseases, such as cardiovascular disease, diabetes, musculoskeletal disorders, and certain cancers. In addition, obesity in childhood is associated with higher odds of obesity, premature death, and disability in adulthood. Over the past 40 years, overweight and obesity rates increased rapidly in China, and excessive weight has become a major public health problem in China [20]. The increasing trend of excessive weight poses detrimental potential health outcomes for the Chinese population in the future. Therefore, strategies involving reducing sedentary behaviors, especially LT-SB, and promoting physical activities are critical in preventing obesity.

Our findings are consistent with previous studies on Chinese children and adolescents, illustrating higher sedentary behaviors than the average Chinese adult [21]. The mean total daily LT-SB time was 3.92 h among children and adolescents, whereas among adults, including older adults, the mean total daily LT-SB time was 2.86 h. Children and adolescents in China may have higher LT-SB times than in other countries [21]. A study covering 43 countries revealed that 39.4% of adolescents spent less than 1 h per day, and 50.6% spent greater than or equal to 1 h per day on LT-SB [22]. However, the study only included students aged 13–15 years, and the LT-SB was also self-reported. Moreover, the sedentary leisure time did not include time spent on homework. Notably, our study discovered that 95.6% spent greater than 1 h per day, with completing homework being the most prominent sedentary behavior. In our study, the LT-SB included time spent completing homework at home. Children spent an average of 1.5 h per day, and adolescents spent an average of 1.4 h per day studying during leisure time, allying with previous findings that found that Chinese children and adolescents spend more time on homework-related LT-SB [23]. High parental expectations and academic pressure may explain the high homework-related LT-SB [24]. Despite the recent “double reduction” policy issued by the Chinese government and the suspension of online and in-person tutoring classes, the surge of online learning during the COVID-19 pandemic may lead to the increased LT-SB time in children and adolescents. Although our results did not find an association between leisure time homework and excess weight, sedentary activity is associated with cardiometabolic disease and mortality. It was suggested that sedentary leisure time may show great effects on the younger population.

The current intervention studies of sedentary behavior in children and adolescents were conducted primarily in schools by incorporating behavioral change techniques and supportive environmental factors [25]. In our research study, a substantial amount of leisure time is spent on homework, and this underlies the significance and urgency of future interventions in nonschool settings, such as at home and within a community. In addition, our study discovered that children aged 6 years and older spent most of their sedentary leisure time watching television or videos, followed by completing homework. Previous research reported the growing trend of screen-based media, occupying active leisure time in children and adolescents [26]. Our study also advocates the importance of promoting healthy behaviors—such as reducing sedentary behavior, performing adequate physical activity, and limiting screen time—in the early stages of child development to avoid unfavorable impacts on adulthood leisure habits.

Our study detects discrepancies in LT-SB compositions between Chinese adults and children. Middle-aged and older adults constitute the largest proportion of participants who watch TV or video during leisure time. In contrast, computer use mainly comprised adolescents and young adults. Children, adolescents, and young adults are also the largest population who spend time online or play video games. The elevated computer-based LT-SB time may be attributed to the social norms of entertainment choices in young Chinese people and the dependence on computers when studying and working [27]. In addition, young people are more accustomed to smart electric devices than middle-aged and older adults. Therefore, younger individuals spend more time on smart electric devices, resulting in increasingly extended periods of computer LT-SB time. Thus, targeted, age-specific, and practical LT-SB guidance and lifestyle interventions are needed to prevent obesity and noncommunicable diseases. Most current interventions for adults are applied in the workplace by installing height-adjustable desks or standing tables [25]. Since the working styles of young adults may influence leisure time sedentariness, future research should consider theory-driven sedentary behavior interventions combined with environmental interventions, such as education and activity-tracking devices [25]. Furthermore, work from home and social isolation during the COVID-19 pandemic increased LT-SB time, further illustrating the importance of interventions in sedentary behavior [28].

In contrast, middle-aged and older adults spend more time than younger adults on other LT-SB, such as television, board games, and chatting, which is in accordance with other studies [23]. Our study found that sedentary computer use in children and young adults was negatively associated with the risk of excessive weight. However, game playing in the middle-aged population and television viewing in older adults were associated with a higher risk of excessive weight. A previous study has shown that watching television has been associated with an increased risk of coronary heart disease, but sitting times on the computer are not related to the risk of coronary heart disease [29]; this may be possibly due to the observation that computer use requires cognitive effort and brain activity (e.g., desk-based office work) [30], whereas watching TV tends to be a passive sedentary behavior, associated with a potentially higher chance of consuming snacks, disordered eating timing, decreased exercise, and increased sleep times during the day [31]. On the other hand, playing games during leisure time may also be accompanied with increased snack consumption. A recent study has reported that time spent watching TV was associated with increased risks of incident dementia and time spent using a computer was associated with decreased risks of incident dementia. Reducing the cognitively passive SB times (i.e., TV) and increasing time spent on cognitively active SB activities (i.e., computer) may be effective behavioral modification targets for reducing the risk of dementia [32].

Older adults are more susceptible to sedentary effects compared to young people due to their fragile health status. Due to physiological and pathological reasons, such as frailty and multifactorial geriatric syndrome and reduced metabolism [33], older adults will also increase their sedentary behavior during leisure time, especially within the context of the COVID-19 epidemic. Furthermore, older adults who spend more time sitting are linked with unhealthy dietary behavior, impairing their weight status [34]. Therefore, if the older adults are unavoidably sedentary during leisure time, community social workers or family caregivers should try to change the type of sedentary and replace passive sedentary behavior with active sedentary behavior, thereby reducing weight gain, cognitive decline, and the risk of cardiovascular disease. Future research may focus on home or community-based active sedentary behavior interventions for older adults.

### Strength and Limitation

The results of this study have many strengths. Firstly, using large, nationally representative survey data with strict quality control ensures the quality of the results. Secondly, this study analyzed the distribution of LT-SB and its impact on excessive weight in different age groups of the Chinese population from 2004 to 2011. Thirdly, we adjusted for lifestyle-related factors, such as socio-demographics and nutritional energy, to reduce the potential bias of the results when analyzing the effect of sedentary lifestyles on excess weight. In addition, we divided LT-SB behaviors into subgroups to provide more detailed evidence for future interventions on excess weight and sedentary behaviors. However, the study also has several limitations. First, since the study analyzed a large national sample, a questionnaire was used to measure LT-SB and physical activity. However, this self-report measure relies heavily on the participant’s mental abilities with respect to making judgments, such as cognitive function, memory recall function, reading ability, and visual ability, and may result in desirability bias [35]. Our study showed that the LT-SB time of older adults was lower than the average of other studies, which may be influenced by self-report and recall bias [36]. Future studies may use objective measures, such as accelerometers, pedometers, or integrated monitors, to yield more accurate measurements. Second, our study focused on leisure-time sedentary behavior and did not analyze sedentary times throughout the day. This is because, compared to work time sedentary behavior and commuting sedentary behavior, leisure-time sedentary behavior is more modifiable.

## 5. Conclusions

In this study, the distribution of Chinese LT-SB and the influence of LT-SB on excessive weight varied by age, with children and adolescents reporting longer LT-SB times compared to adults. The impact of screen-based LT-SB on excessive weight is greater in middle-aged and older adults than in younger adults, adolescents, and children. The findings of this study provide a more comprehensive understanding of the target population for public health policies and ascertain the urgency and priority of research on leisure-time sedentary behavior in China.

## Figures and Tables

**Table 1:** Association between leisure-time sedentary behaviors and excessive weight among the Chinese population, CHNS 2004-2011.

Variables	OR (95% C.I.)				
	6–11 y	12–17 y	18–44 y	45–60 y	>=60 y
Excessive Weight (Overweight and Obesity)					
Watch TV or video (h/d)	1.030 (0.817–1.299)	1.087 (0.955–1.237)	0.999 (0.974–1.024)	1.014 (0.990–1.039)	1.065 (1.035–1.095)
Computer use (h/d)	0.200 (0.053–0.755)	1.108 (0.915–1.343)	0.921 (0.881–0.962)	0.980 (0.902–1.065)	0.907 (0.781–1.054)
Play online or video games (h/d)	1.033 (0.630–1.684)	0.768 (0.563–1.047)	0.983 (0.924–1.045)	1.122 (1.005–1.253)	1.196 (0.975–1.469)
Do homework (h/d)	0.838 (0.640–1.099)	0.940 (0.819–1.078)	-	-	-
Reading (h/d)	1.080 (0.741–1.574)	0.910 (0.736–1.126)	0.970 (0.916–1.028)	1.059 (0.982–1.142)	0.979 (0.906–1.058)

Note: OR: odds ratio; CI: confidence interval; h/d: hours per day. For the 6–11- and 12–17-year-old groups, sex, energy intake, physical activity, region (in urban and rural areas), and survey year were adjusted. For the above 18-year-old group, sex, energy intakes, physical activity, region (in urban and rural areas), survey year, educational level, and smoking status were adjusted.

## Data Availability

The China Health and Nutrition Survey datasets is available at http://www.cpc.unc.edu/projects/china.

## Supplementary Materials

The following supporting information can be downloaded at: https://www.mdpi.com/article/10.3390/nu15020424/s1, Table S1. Characteristics of the study participants according to sex; Table S2. Sample size for leisure-time sedentary behaviors in the Chinese population by socio-demographic and lifestyle characteristics, CHNS 2004-2011; Table S3. Association between total leisure-time sedentary behavior and excessive weight among the Chinese population, CHNS 2004-2011.

## References

[1] Li F., Mao L., Chen P. (2019). Physical activity and prevention of chronic disease in Chinese youth: A public health approach. J. Sport Health Sci..

[2] Tian Y., Jiang C., Wang M., Cai R., Zhang Y., He Z., Wang H., Wu D., Wang F., Liu X. (2016). BMI, leisure-time physical activity, and physical fitness in adults in China: Results from a series of national surveys, 2000–2014. Lancet Diabetes Endocrinol..

[3] Mandviwala T., Khalid U., Deswal A. (2016). Obesity and Cardiovascular Disease: A Risk Factor or a Risk Marker?. Curr. Atheroscler. Rep..

[4] Calle E.E., Kaaks R. (2004). Overweight, obesity and cancer: Epidemiological evidence and proposed mechanisms. Nat. Rev. Cancer.

[5] Bjerregaard L.G., Baker J.L. (2018). Change in Overweight from Childhood to Early Adulthood and Risk of Type 2 Diabetes. N. Engl. J. Med..

[6] Monda V., La Marra M., Perrella R., Caviglia G., Iavarone A., Chieffi S., Messina G., Carotenuto M., Monda M., Messina A. (2017). Obesity and brain illness: From cognitive and psychological evidences to obesity paradox. Diabetes Metab. Syndr. Obes..

[7] Van Dammen L., Wekker V., de Rooij S.R., Groen H., Hoek A., Roseboom T.J. (2018). A systematic review and meta-analysis of lifestyle interventions in women of reproductive age with overweight or obesity: The effects on symptoms of depression and anxiety. Obes. Rev..

[8] Lavie C.J., Ozemek C., Carbone S., Katzmarzyk P.T., Blair S.N. (2019). Sedentary Behavior, Exercise, and Cardiovascular Health. Circ. Res..

[9] Ng S.W., Popkin B.M. (2012). Time use and physical activity: A shift away from movement across the globe. Obes. Rev..

[10] Bull F.C., Al-Ansari S.S., Biddle S., Borodulin K., Buman M.P., Cardon G., Carty C., Chaput J.-P., Chastin S., Chou R. (2020). World Health Organization 2020 guidelines on physical activity and sedentary behaviour. Br. J. Sport. Med..

[11] Felez-Nobrega M., Raine L.B., Haro J.M., Wijndaele K., Koyanagi A. (2020). Temporal trends in leisure-time sedentary behavior among adolescents aged 12–15 years from 26 countries in Asia, Africa, and the Americas. Int. J. Behav. Nutr. Phys. Act..

[12] Yang L., Cao C., Kantor E.D., Nguyen L.H., Zheng X., Park Y., Giovannucci E.L., Matthews C.E., Colditz G.A., Cao Y. (2019). Trends in Sedentary Behavior Among the US Population, 2001–2016. JAMA.

[13] Stamatakis E., Gale J., Bauman A., Ekelund U., Hamer M., Ding D. (2019). Sitting Time, Physical Activity, and Risk of Mortality in Adults. J. Am. Coll. Cardiol..

[14] Loyen A., Clarke-Cornwell A.M., Anderssen S.A., Hagströmer M., Sardinha L.B., Sundquist K., Ekelund U., Steene-Johannessen J., Baptista F., Hansen H.B. (2017). Sedentary Time and Physical Activity Surveillance Through Accelerometer Pooling in Four European Countries. Sport. Med..

[15] Fu W., Zhao S., Zhang Y., Chai P., Goss J. (2018). Research in health policy making in China: Out-of-pocket payments in Healthy China 2030. BMJ.

[16] Zhang B., Zhai F.Y., Du S.F., Popkin B.M. (2014). The China Health and Nutrition Survey, 1989–2011. Obes. Rev..

[17] Popkin B.M., Du S., Zhai F., Zhang B. (2010). Cohort Profile: The China Health and Nutrition Survey--monitoring and understanding socio-economic and health change in China, 1989–2011. Int. J. Epidemiol..

[18] Liu Z., Xing B., Xue Z.Z. (2004). Group of China Obesity Task Force. Body mass index reference norm for screening overweight and obesity in Chinese children and adolescents. Zhonghua Liuxingbingxue Zazhi.

[19] Blüher M. (2019). Obesity: Global epidemiology and pathogenesis. Nat. Rev. Endocrinol..

[20] Pan X.F., Wang L., Pan A. (2021). Epidemiology and determinants of obesity in China. Lancet Diabetes Endocrinol..

[21] Silva D.A.S., Chaput J.P., Katzmarzyk P.T., Fogelholm M., Hu G., Maher C., Tremblay M.S. (2018). Physical Education Classes, Physical Activity, and Sedentary Behavior in Children. Med. Sci. Sport. Exerc..

[22] Vancampfort D., Stubbs B., Mugisha J., Firth J., Van Damme T., Smith L., Koyanagi A. (2019). Leisure-time sedentary behavior and suicide attempt among 126,392 adolescents in 43 countries. J. Affect. Disord..

[23] Rawlings G.H., Williams R.K., Clarke D.J., English C., Fitzsimons C., Holloway I., Lawton R., Mead G., Patel A., Forster A. (2019). Exploring adults’ experiences of sedentary behaviour and participation in non-workplace interventions designed to reduce sedentary behaviour: A thematic synthesis of qualitative studies. BMC Public Health.

[24] Li M., Xue H., Wang W., Wang Y. (2017). Parental Expectations and Child Screen and Academic Sedentary Behaviors in China. Am. J. Prev. Med..

[25] Blackburn N.E., Wilson J.J., McMullan I.I., Caserotti P., Giné-Garriga M., Wirth K., Tully M.A. (2020). The effectiveness and complexity of interventions targeting sedentary behaviour across the lifespan: A systematic review and meta-analysis. Int. J. Behav. Nutr. Phys. Act..

[26] Auhuber L., Vogel M., Grafe N., Kiess W., Poulain T. (2019). Leisure Activities of Healthy Children and Adolescents. Int. J. Environ. Res. Public Health.

[27] Tremblay M.S., Colley R.C., Saunders T.J., Healy G.N., Owen N. (2010). Physiological and health implications of a sedentary lifestyle. Appl. Physiol. Nutr. Metab..

[28] Xiao Y., Becerik-Gerber B., Lucas G., Roll S.C. (2021). Impacts of Working From Home During COVID-19 Pandemic on Physical and Mental Well-Being of Office Workstation Users. J. Occup. Environ. Med..

[29] Kim Y., Yeung S.L.A., Sharp S.J., Wang M., Jang H., Luo S., Brage S., Wijndaele K. (2022). Genetic susceptibility, screen-based sedentary activities and incidence of coronary heart disease. BMC Med..

[30] Hallgren M., Nguyen T.T., Owen N., Stubbs B., Vancampfort D., Lundin A., Dunstan D., Bellocco R., Lagerros Y.T. (2020). Cross-sectional and prospective relationships of passive and mentally active sedentary behaviours and physical activity with depression. Br. J. Psychiatry.

[31] Hallgren M., Dunstan D.W., Owen N. (2020). Passive versus mentally active sedentary behaviors and depression. Exerc. Sport Sci. Rev..

[32] Raichlen D.A., Klimentidis Y.C., Sayre M.K., Bharadwaj P.K., Lai M.H.C., Wilcox R.R., Alexander G.E. (2022). Leisure-time sedentary behaviors are differentially associated with all-cause dementia regardless of engagement in physical activity. Proc. Natl. Acad. Sci. USA.

[33] Da Silva Coqueiro R., de Queiroz B.M., Oliveira D.S., das Merces M.C., Oliveira Carneiro J.A., Pereira R., Fernandes M.H. (2017). Cross-sectional relationships between sedentary behavior and frailty in older adults. J. Sport. Med. Phys. Fit..

[34] Hsueh M.C., Rutherford R., Huang Y.H., Chang Chien H.Y., Chang C.H., Park J.H., Liao Y. (2019). Are Older Adults without a Healthy Diet Less Physically Active and More Sedentary?. Nutrients.

[35] Cleland C., Ferguson S., Ellis G., Hunter R.F. (2018). Validity of the International Physical Activity Questionnaire (IPAQ) for assessing moderate-to-vigorous physical activity and sedentary behaviour of older adults in the United Kingdom. BMC Med. Res. Methodol..

[36] Copeland J.L., Ashe M.C., Biddle S.J.K., Brown W.J., Buman M.P., Chastin S., Gardiner P.A., Inoue S., Jefferis B.J., Oka K. (2017). Sedentary time in older adults: A critical review of measurement, associations with health, and interventions. Br. J. Sport. Med..

