# Supplementary material for: Association between Sedentary Behavior during Leisure Time and Excessive Weight in Chinese Children, Adolescents, and Adults
Source: Nutrients. 2023 Jan 13;15(2):424. doi: 10.3390/nu15020424 (PMC9867297; doi:10.3390/nu15020424)
Supplement: Supplementary file 1 [file nutrients-15-00424-s001.zip › nutrients-2116899-supplementary.pdf]

Table S1. Characteristics of the study participants according to sex

	Male	Female
**Age**	47.06 ± 16.53	47.51 ± 16.11
**BMI**	23.62 ± 3.19	23.71 ± 3.39
**Leisure-time PA (hours/day)**	0 (0-3.0)	0 (0-3.0)
**Leisure-time SB (hours/day)**		
Watch TV or video	2.0 (0-15.29)	2.0 (0-15.14)
Computer use	0 (0-13.0)	0 (0-10.29)
Play online or video games	0 (0-10.0)	0 (0-6.0)
Do Homework	0 (0-7.71)	0 (0-12.14)
Reading	0 (0-13.0)	0 (0-11.43)
**Total leisure-time sedentary time (hours/day)**	2.52 (0-15.86)	2.29 (0-15.49)
**Energy intake (kkcal/day)**	2.35 (0.08-6.20)	1.98 (0.19-6.19)

Results are presented as mean ± SD, median (range)

Table S2. Sample Size for Leisure-time sedentary behaviors in the Chinese population by sociodemographic and lifestyle characteristics, CHNS 2004-2011

**Variables**	**No. of Participants by Age group, n (%)**				
	**6- 11**	**12 - 17**	**18 - 44**	**45 - 59**	**≥ 60**
**Overall**	513 (100)	1286 (100)	13494 (100)	12492 (100)	8384 (100)
**Sex**					
Male	308 (58.6)	657 (51.1)	6350 (47.1)	5985 (47.9)	4028 (48.0)
Female	205 (42.2)	629 (48.9)	7144 (52.9)	6507 (52.1)	4356 (52.0)
**Weight status**					
Normal	410 (79.9)	1102 (85.7)	8617 (63.9)	6458 (51.7)	4583 (54.7)
**Excessive weight**	103 (20.1)	184 (14.3)	4877 (36.1)	6034 (48.3)	3801 (45.3)
**Educational level**					
<High school	-	-	8581 (63.6)	9005 (72.1)	6953 (82.9)
High school	-	-	3312 (24.5)	2897 (23.2)	983 (11.7)
>High school	-	-	1580 (11.7)	580 (4.6)	426 (5.1)
Missing	-	-	21 (0.2)	10 (0.1)	22 (0.3)
**Region**					
Rural	311 (60.6)	792 (61.6)	9157 (67.9)	8170 (65.4)	4715 (56.2)
Urban	202 (39.4)	494 (38.4)	4337 (32.1)	4322 (34.6)	3669 (43.8)
**Leisure-time PA**					
< 60 min/week	339 (66.1)	801 (62.3)	11876 (88.0)	11065 (88.6)	7238 (86.3)
≥ 60 min/week	24 (4.7)	62 (4.8)	200 (1.5)	104 (0.8)	43 (0.5)
≥ 150 min/week	63 (12.3)	136 (19.6)	497 (3.7)	283 (2.3)	251 (3.0)
≥ 300 min/week	87 (17.0)	287 (22.3)	923 (6.8)	1040 (8.3)	852 (10.2)
**Smoking status**					
Never	-	1244 (97.7)	9605 (71.2)	8211 (65.7)	5670 (67.6)
Former	-	7 (0.5)	244 (1.8)	434 (3.5)	683 (8.1)
Current	-	27 (2.1)	3632 (26.9)	3835 (30.7)	2025 (24.2)
Missing		8 (0.6)		12 (0.1)	
**Leisure-time SB**					
**Watch TV or video**					
≤ 1.0 hour/day	197 (38.4)	657 (51.1)	3254 (24.1)	2681 (21.5)	2137 (25.5)
1.1-2.0 hours/day	192 (37.4)	495 (38.5)	5026 (37.2)	4729 (37.9)	2824 (33.7)
> 2.0 hours/day	124 (24.2)	134 (10.4)	5214 (38.6)	5082 (40.7)	3423 (40.8)
**Computer use**					
< 1.0 hour/day	488 (95.1)	1069 (83.1)	10913 (80.9)	11846 (94.8)	8232 (98.2)
≥ 1.0 hours/day	25 (4.9)	217 (16.9)	2581 (19.1)	646 (5.2)	152 (1.8)

**Play online or video games**					
< 1.0 hour/day	476 (92.8)	1170 (91.0)	12026 (89.1)	12077 (96.7)	8293 (98.9)
≥ 1.0 hours/day	37 (7.2)	116 (9.0)	1468 (10.9)	415 (3.3)	91 (1.1)
**Do Homework**					
< 1.0 hour/day	167 (32.6)	523 (40.7)	-	-	-
≥ 1.0 hours/day	346 (67.4)	763 (59.3)	-	-	-
**Reading**					
< 1.0 hour/day	389 (75.8)	1005 (78.1)	12026 (89.1)	11137 (89.2)	7147 (85.2)
≥ 1.0 hours/day	124 (24.2)	281 (21.9)	1468 (10.9)	1355 (10.8)	1237 (14.8)
**Survey year**					
2004	100 (19.5)	403 (31.3)	3345 (24.8)	2627 (21.0)	1432 (17.1)
2006	106 (20.7)	271 (21.1)	3209 (23.8)	2620 (21.0)	1677 (20.0)
2009	101 (19.7)	244 (19.0)	3057 (22.7)	3055 (24.5)	2120 (25.3)
2011	206 (40.2)	368 (28.6)	3883 (28.8)	4190 (33.5)	3155 (37.6)

Note: Excessive weight: combined overweight and obesity; PA: physical activity; SB: sedentary behavior.

Table S3. Association of total leisure-time sedentary time with excessive weight among the Chinese population, CHNS 2004-2011

Variables	OR (95% C.I.)	
	**Male**	**Female**
**Age, years**	1.009 (1.006-1.011)	1.024 (1.022-1.026)
**Educational level**		
< high school	1 [Reference]	1 [Reference]
high school	1.349 (1.249-1.458)	0.775 (0.711-0.845)
> high school	1.420 (1.261-1.599)	0.530 (0.456-0.616)
**Region**		
Rural	1 [Reference]	1 [Reference]
Urban	1.376 (1.281-1.478)	1.046 (0.976-1.121)
**Survey year**		
2004	1 [Reference]	1 [Reference]
2006	1.084 (0.985-1.193)	0.964 (0.879-1.057)
2008	1.262 (1.150-1.385)	1.012 (0.925-1.107)
2011	1.415 (1.296-1.545)	1.203 (1.105-1.309)
**Leisure-time PA (hours/day)**	0.993 (0.984-1.003)	1.007 (0.997-1.017)
**Energy intake (kkcal/day)**	1.171 (1.126-1.218)	1.142 (1.095-1.192)
**Smoking status**		
Never	1 [Reference]	1 [Reference]
Former	1.170 (1.033-1.325)	1.028 (0.645-1.636)
Current	0.830 (0.777-0.887)	0.860 (0.726-1.019)
**Total leisure-time sedentary time**		
≤ 1 hour/day	1 [Reference]	1 [Reference]
≤ 2 hours/day	1.214 (1.095-1.346)	1.185 (1.084-1.295)
> 2 hours/day	1.166 (1.059-1.284)	1.130 (1.038-1.230)

Note: PA: physical activity; OR: odds ratio; CI: confidence interval; sex, energy intakes, physical activity, region (in urban and rural areas), and survey year, and educational level, and smoking status were adjusted for.
